# Direct Binding and Regulation by Fur and HapR of the Intermediate Regulator and Virulence Factor Genes Within the ToxR Virulence Regulon in *Vibrio cholerae*

**DOI:** 10.3389/fmicb.2020.00709

**Published:** 2020-04-17

**Authors:** He Gao, Jingyun Zhang, Jing Lou, Jie Li, Qin Qin, Qiannan Shi, Yiquan Zhang, Biao Kan

**Affiliations:** ^1^State Key Laboratory for Infectious Disease Prevention and Control, National Institute for Communicable Disease Control and Prevention, Chinese Center for Disease Control and Prevention, Beijing, China; ^2^School of Medicine, Jiangsu University, Zhenjiang, China

**Keywords:** *Vibrio cholerae*, toxin coregulated pilus, cholera toxin, HapR, Fur

## Abstract

Cholera toxin (CT) and toxin coregulated pilus (TCP, TcpA is the major subunit) are two major virulence factors of *Vibrio cholerae*, both of which play critical roles in developing severe diarrhea in human. Expression of CT and TCP is under the tight control of the regulatory cascade known as the ToxR virulence regulon, which is composed of three regulators ToxR, TcpP, and ToxT. Besides, their expression is also regulated by the quorum sensing (QS) master regulator HapR and the regulatory protein Fur. Though transcription of *tcpP*, *toxT*, and/or *tcpA* are reported to be regulated by HapR and Fur, to date there are no studies to verify their direct regulations. In the present study, we showed that HapR directly repress the transcription of *tcpP* and *tcpA* by binding to their promoter regions, and possibly repress *toxT* transcription in an indirect manner. Fur directly activated the transcription of *tcpP*, *toxT*, and *tcpA* by binding to their promoters. Taking account of the sequential expression of *hapR*, *fur*, *tcpP*, *toxT*, and *tcpA* in the different growth phases of *V. cholerae*, we deduce that at the early mid-logarithmic growth phase, Fur binds to the promoters of *tcpP*, *toxT*, and *tcpA* to activate their transcription; while at the later mid-logarithmic growth phase, HapR can bind to the promoters of *tcpP* and *tcpA* to repress their transcription. Our study reveals the new recognition in the virulence regulatory pathways in *V. cholerae* and suggests the complicated and subtle regulation network with the growth density dependence.

## Introduction

*Vibrio cholerae* is a Gram-negative bacterium that naturally inhabits salty coastal waters and estuaries (Clemens et al., 2017), and some are the causative agent of cholera. Two virulence factors, cholera toxin (CT) and toxin coregulated pilus (TCP), are considered the most closely connected to cholera. CT, encoded by the *ctxAB* operon in the *V. cholerae* lysogenic phage CTXΦ, is an AB_5_ toxin that consists of a single catalytic A-subunit and a pentamer of B-subunits (Merritt and Hol, 1995). It can enhance the concentration of intracellular cyclic AMP, which then causes an imbalance in electrolyte transport across the intestinal epithelial cell membrane, resulting in the secretion of water and electrolytes into the bowel accompanied by severe watery diarrhea, which may lead to death without timely treatment (Clemens et al., 2017). TCP, the subunit of which is encoded by *tcpA* (in the *tcpABQCRDSTEF* operon), is essential for colonization of *V. cholerae* in the small intestine at the early stage of infection (Kirn et al., 2000). It also functions as the receptor for the CTXΦ (Waldor and Mekalanos, 1996).

The expression of TCP and CT is tightly regulated by a regulatory cascade, referred to as the ToxR virulence regulon (Childers and Klose, 2007). Under virulence inducing growth conditions, ToxR cooperates with TcpP to bind to the promoter region of *toxT* to activate its transcription, and ToxT, in turn, activates the transcription of *ctxAB* and *tcpA* (DiRita et al., 1991; Krukonis et al., 2000; Goss et al., 2013). ToxR alone also can directly activate *ctxAB* transcription in the presence of bile acids (Hung and Mekalanos, 2005). While under non-inducing growth conditions, TcpP and ToxT are proteolytically degraded in order to terminate virulence gene expression (Matson and DiRita, 2005; Abuaita and Withey, 2011). The genes for TCP and CT production are also regulated by quorum sensing (QS) (Miller et al., 2002; Zhu et al., 2002), a cell-to-cell communication process that bacteria use to monitor their cell density by detecting the extracellular concentration of autoinducers (AIs), the signaling molecules (Ball et al., 2017). In vibrios, AphA and LuxR orthologs (referred to as the HCD master regulators, HMRs) represent the terminal master regulator of QS operating at low cell density (LCD) and high cell density (HCD), respectively (Lu et al., 2018). AphA, which has interaction with AphB, binds to the promoter of *tcpPH* to activate its transcription (Kovacikova et al., 2004). HapR (the homologous protein of LuxR) represses the transcription of *tcpPH* via binding and repression of *aphA* transcription (Kovacikova and Skorupski, 2002). The global regulator cAMP-CRP represses *tcpPH* transcription via its ability to influence AphA- and AphB-dependent transcriptional activation of *tcpPH*. This is because the cAMP-CRP binding site is completely within the binding sites of AphA and AphB (Kovacikova and Skorupski, 2001). H-NS also has roles in silencing the expression of TCP and CT by binding and repression of *ctx*, *tcp*, and *toxT* promoters (Nye et al., 2000; Stonehouse et al., 2011). In addition, the ferric uptake regulator Fur seems to have positive regulatory activity on TCP production, because deletion of *fur* repressed *tcp* transcription and exhibited very weak autoagglutination, one indicator of the capacity of *V. cholerae* infection *in vivo* (Mey et al., 2005).

Although HapR repression of TCP and CT via repression of AphA has been demonstrated, whether HapR can directly regulate the genes within the ToxR virulence regulon or not, needs to be further investigated. In addition, the mechanisms of the Fur-dependent activation of TCP expression are also unclear. Moreover, transcription of *fur* was under the direct control of HapR, and HapR coordinates with Fur to regulate *hlyA* transcription (Gao et al., 2018), suggesting Fur integrated into QS to co-regulate gene expression in *V. cholerae*.

**FIGURE 1:**
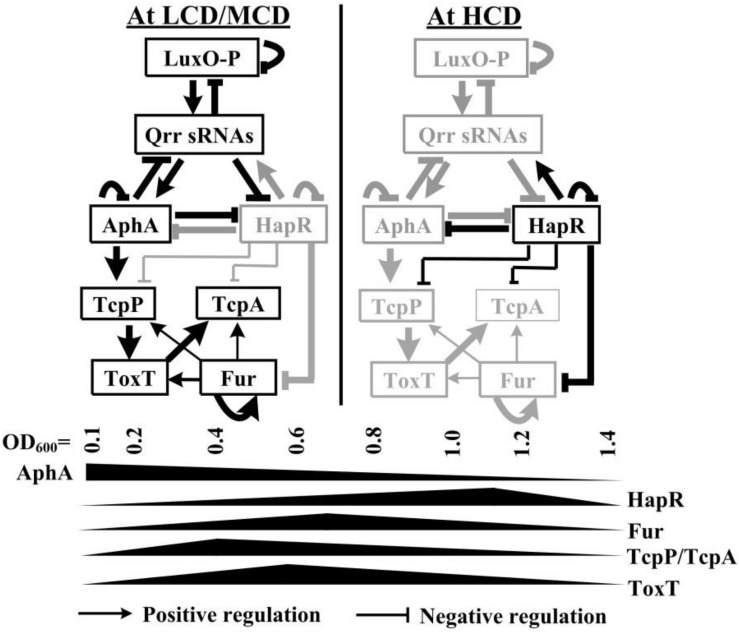
Regulatory model. The regulatory relationships between LuxO, Qrrs, AphA, and HapR (or its homologous protein) have been previously reported in vibrios (Henke and Bassler, 2004; Tu et al., 2010; Rutherford et al., 2011; Sun et al., 2012b; Zhang et al., 2012). AphA and HapR are the bottom master regulators of QS operating at LCD and HCD, respectively. Fur coordinates with HapR to tightly regulate *tcpP*, *toxT*, and *tcpA*, leading to the cell density transcriptional manner of expression of these genes. HapR repression of Fur (Gao et al., 2018), AphA activation of *tcpP* (Kovacikova and Skorupski, 2001; Kovacikova et al., 2004), TcpP activation of *toxT* (Krukonis et al., 2000), autoregulation of Fur (Lee et al., 2007), and ToxT activation of *tcpA* (DiRita et al., 1991) were reported by other researchers. LCD, low cell density; MCD, medium cell density; HCD, high cell density.

In the present study, we showed that transcription of *tcpP*, *toxT*, and *tcpA* were all cell-density dependent, which may be due to the coordinated regulation of Fur and HapR (Figure 1). At the OD_600_ value of about 0.7, Fur binds to the promoters of *tcpP*, *toxT*, and *tcpA* to activate their transcription; while at the OD_600_ value of about 1.0, the QS regulator HapR directly represses the transcription of *tcpP* and *tcpA*, but it indirectly represses *toxT* transcription. The data enriched the regulatory networks that control the expression of virulence determinants in *V. cholerae*, which promotes a deeper understanding of the pathogenic mechanisms of the pathogen.

## Materials and Methods

### Bacterial Strains and Cultural Conditions

*Vibrio cholerae* El Tor serogroup O1 strain C7258 (Peru, 1991) was used as the derivative (wild type, WT). Non-polar *fur* and *hapR* single-gene deletion mutants Δ*fur* and Δ*hapR* derived from the WT strain were constructed in our previous study (Gao et al., 2018). The deletion mutant of *lacZ* (designated as Δ*lacZ*) was constructed from WT by homologous recombination using the suicide plasmid pWM91, which was similarly performed as previously described (Wu et al., 2015). All primers used in the present work are listed in Table 1.

**TABLE 1:** Oligonucleotide primers used in this study.

Target	Primers (forward/reverse, 5′–3′)
**Construction of mutants**	
*fur*	CGGGATCCTTCGTGTAAGGCAGCAGTAATC/CAGAGCGTAAAGCCTATGGATACTTTCCTGTTGATGTTC
	GAACATCAACAGGAAAGTATCCATAGGCTTTACGCTCTG/GGACTAGTAGATGAAGATGGTGTGGGAAAC
	CGGGATCCTTCGTGTAAGGCAGCAGTAATC/GGACTAGTAGATGAAGATGGTGTGGGAAAC
*hapR*	GCGGGATCCCCAGCAATACATCTTTACC/GTGCTGCCCAAGAAAAGGGGTATATCCTTGCC
	GGCAAGGATATACCCCTTTTCTTGGGCAGCAC/GCGACTAGTAACTCACCAAAACCTTC
	GCGGGATCCCCAGCAATACATCTTTACC/GCGACTAGTAACTCACCAAAACCTTC
*lacZ*	GCGGGATCCCACGGAGGGAAGGGTAAA/CCTTAAGGCTCTCTGGCCCCTCAAGCCGAGGAGTAAAG
	CTTTACTCCTCGGCTTGAGGGGCCAGAGAGCCTTAAGG/GGACTAGTCAGCCCAGACAGTGAAGG
	GCGGGATCCCACGGAGGGAAGGGTAAA/GGACTAGTCAGCCCAGACAGTGAAGG
**Protein expression**	
*fur*	GCGGGATCCATGTCAGACAATAACCAAG/GCGAAGCTTTTATTTCTTCGGCTTGTGAG
*hapR*	GCGGGATCCATGGACGCATCAATCGAAAAAC/GCGAAGCTTCTAGTTCTTATAGATACACAG
**qPCR**	
*tcpP*	GCACAAGATCCAATGAAGCC/CTGGTTCTTTTGATTGCCTGAG
*tcpA*	TGGTCTCAGCGGGTGTTG/CATTTGCGTTTGCGGTAGC
*toxT*	TTTTCAGGGTTCTTCTCG/ACAAATATCTGCCCAACG
*toxR*	TTTGTTTGGCGAGAGCAAGG/TCTTCTTCAACCGTTTCCACTC
*recA*	AAGATTGGTGTGATGTTTGGTA/CACTTCTTCGCCTTCTTTGA
**Primer extension**	
*tcpA*	/CCAGAACAATGATTACTTC-HEX
**Luminescence assay**	
*tcpP*	GCGGAGCTCGTGCCTGCTGAGAACTAA/GCGGGATCCCAAAGGTTATCGGGAAAT
*tcpA*	GCGGAGCTCTCCCGACTACTCAGAAAG/GCGGGATCCATTTATATAACTCCACC
*toxT*	GCGGAGCTCGTGAATGTTGGTGG/GCGGGATCCTGCGTTCTACTCTG
*toxR*	GCGGAGCTCTCCGCACCGTCACCGC/GCGGGATCCCTAATGTCCCAGTATC
*fur*	GCGGAGCTCGCATCAAGGCATAAACGG/GCGACTAGTATACTTTCCTGTTGATGTTC
*hapR*	GCGGAGCTCCCAGCAATACATCTTTACC/GCGACTAGTTGAGGCGATAGCCGAGTT
**DNase I footprinting**	
*tcpP*	GTAAAACGACGGCCAGTCAGGAAAGATAATGTAACC/CAGGAAACAGCTATGACGTGTACCAATCAGCCTTT
	GTAAAACGACGGCCAGTGTGCCTGCTGAGAACTAA/CAGGAAACAGCTATGACGGGCTTTTTTTAACTTTG
*tcpA*	GTAAAACGACGGCCAGTTCCCAATTGGTTGGCTC/CAGGAAACAGCTATGACCATATTTATATAACTCCACC
*toxT*	GTAAAACGACGGCCAGTCAGGTCGATTTCTTAC/CAGGAAACAGCTATGACTTCCACTATCTATCC
	GTAAAACGACGGCCAGTCAGGTCGATTTCTTAC/CAGGAAACAGCTATGACCCTTAAACTGCACATC
*toxR*	GTAAAACGACGGCCAGTTCCGCACCGTCACCGC/CAGGAAACAGCTATGACCCAATATGACTCATCG
M13	FAM-GTAAAACGACGGCCAGT/CAGGAAACAGCTATGAC-HEX

All strains were maintained at −80°C in LB broth [1% tryptone (Oxoid), 0.5% yeast extract (Oxoid), and 1% NaCl (Merck Millipore)] containing 30% glycerol (v/v). Unless stated otherwise, *V*. *cholerae* strains were cultured with AKI [1.5% Bacto peptone (BD Biosciences), 0.4% yeast extract (Oxoid), 0.5% NaCl (Merck Millipore), and 0.3% NaHCO_3_ (Merck Millipore)] of the TCP-induced conditions as previously described (Iwanaga et al., 1986). When appropriate, the culture medium was supplemented with 100 μg/ml ampicillin or 50 μg/ml kanamycine.

### Competition Assay

*In vivo* competition assay was performed as previously described (Tamayo et al., 2010). The Δ*lacZ*, Δ*fur* and Δ*hapR* strains were grown overnight on LB agar containing the appropriate antibiotics at 37°C. For each strain approximately 10 colonies were resuspended in 1 ml phosphate-buffered saline (PBS). The strains were mixed 1:1 and adjusted to a final concentration of approximately 10^6^CFU/ml. Five-day-old CD-1 infant mice were used as the infection model, and each mouse was intragastrically inoculated with 100 μl (about 10^5^ CFU) of the mixture (Tamayo et al., 2010). Mice were sacrificed at 20 h post-inoculation, and the bacterial cells in the intestines were enumerated on LB agar plates containing 40 μg/ml of 5-bromo-4-chloro-3-indolyl-β-D-galactopyranoside (*X*-gal) to differentiate the Δ*lacZ* and the regulatory gene mutant colonies and to determine the input ratios and bacterial titers. *In vitro* competitions were performed in parallel to calculate the input ratios by inoculating 2 μl of the mix into 1 ml LB and incubating overnight at 37°C with aeration. The competition index (CI) of the input and the output were calculated as the blue/white ratio of the mutant/Δ*lacZ*.

This work was performed in strict accordance with animal protocols approved by the Ethics Committee of the National Institute for Communicable Disease Control and Prevention, China CDC.

### Quantitative PCR (qPCR)

Extraction of bacterial total RNAs, generation of cDNAs, and operation of qPCR were performed as previously described (Gao et al., 2011). The relative mRNA levels were determined based on the standard curve of *recA* (reference gene) expression for each RNA preparation. The qPCR assay was performed with at least three independent biological samples.

### Luminescence Assay

For the luminescence assay (Xu et al., 2010; Gao et al., 2018), the promoter DNA region of each target gene was cloned into the pBBRlux harboring a promoterless *luxCDABE* reporter gene and a chloramphenicol resistance gene. The recombinant plasmid was transferred into WT and each mutant strain, respectively. Strains transformed with recombinant plasmids were cultivated in AKI-inducing conditions and harvested at the required cell densities. The luminescence was measured using an Infinite^®^ 200 Pro NanoQuant (Tecan, Switzerland). The *lux* activity was calculated as light units/OD_600_.

### DNase I Footprinting Assay

The recombinant proteins His-Fur and His-HapR were expressed using the pET28a plasmid and the *Escherichia coli* BL21λDE3 cells (Kleber-Janke and Becker, 2000; Gao et al., 2008). The purified recombinant proteins were concentrated to a final concentration of 0.3 to 0.6 mg/ml. DNase I footprinting and sequencing assays were carried out as previously described (Gao et al., 2017; Zhang et al., 2017b). Briefly, the FAM (or HEX)-labeled DNA probes were incubated with the increasing amounts of His-tagged protein, and digested by the optimized RQ1 RNase-Free DNase I (Promega), and then analyzed using an ABI 3500XL DNA Genetic analyzer with GeneMarker software 2.2, while the DNA sequencing products were surveyed with Sequence Scanner software v1.0.

### Primer Extension Assay

The primer extension assay was essentially performed as previously described (Gao et al., 2018). Twelve micrograms of total RNA was annealed with 1 pmol of 5′- HEX-labeled reverse primer to generate cDNAs using the Primer Extension System (Promega), and the products of primer extension were then analyzed using the ABI 3500XL DNA Genetic analyzer.

### Experimental Replicates and Statistical Methods

The competition assay was done at least three independent times with similar results. The data of DNase I footprinting and primer extension were done at least two independent times. The luminescence assay and qPCR were performed with at least three independent bacterial cultures, and the values were expressed as the mean ± standard deviation (SD). Paired Student’s *t-*test was used to calculate significant differences, *P* < 0.01 was considered to indicate statistical significance.

## Results

### Binding Sites of HapR/Fur Were Predicted Within the Regulatory Regions of *tcpP*, *tcpA*, and *toxT*

Toxin coregulated pilus production is under the control of a tightly regulated signaling cascade composed of ToxR, TcpP/H, and ToxT (Childers and Klose, 2007). It has been reported that HapR represses *tcpA* transcription through binding and repression of *aphA* (Kovacikova and Skorupski, 2002), however, it remains unknown whether HapR can directly repress *tcpA* or not. Here, the 700 bp upstream regions of *toxR*, *tcpP*, *toxT*, and *tcpA* were retrieved from the genome sequence of strain C7258, and the DNA binding box of the HMRs (TATTGATAAA-TTTATCAATA) in vibrios (Zhang et al., 2012) was used to statistically predict the presence of HMRs box-like sequences within the above target promoter sequences by using the *matrix-scan* tool^1^. The higher weighted score for the target gene represented the higher probability of direct protein and DNA sequence association. With the weight score cut-off of 6.0, the HMRs box-like sequences were predicted for *tcpP* and *tcpA*, but not for *toxR* and *toxT* (Table 2), suggesting the possible direct binding of HapR on the promoter regions of these two genes.

**TABLE 2:** Predicted HMRs/Fur box-like sequences within target promoters.

		Fur box-like sequence			HMRs box-like sequence		
Operon	First gene	Position^&^	Sequence	Score	Position^&^	Sequence	Score
*toxRS*	*toxR*	NA	NA	NA	NA	NA	NA
	*toxT*	D-611…-593	AATGAAATTTATCCTCATA	8.9	NA	NA	NA
*tcpPH*	*tcpP*	D-459…-441	AATTATTTTTTTTATCATT	9.4	R-71…-52	TTTTAATATAATTATTTGCA	7.7
*tcpA-F*	*tcpA*	D-273…-255	AACGCATTTTATTTGCATT	7.0	D-125…-106	AAAAATGATATCTGTCAATT	6.1

*^&^‘D’ indicates the direct sequence while ‘R’ the reverse one; minus numbers denote the nucleotide positions upstream of indicated genes; ‘NA’ represents ‘not applicable.’*

We considered that Fur may also possibly to regulate the expression or assembly of TCP, since the *fur* mutant exhibited reduced TCP expression and weak autoagglutination (Mey et al., 2005). In our study, the Fur binding box (Mey et al., 2005; Davies et al., 2011) was used as well for prediction of Fur box-like sequences within the promoter regions of *toxR*, *tcpP*, *toxT*, and *tcpA*. Fur box-like sequences were predicted from each promoter of *toxT*, *tcpP*, and *tcpA*, but none was detected for the *toxR* promoter (Table 2). Expression of Fur itself was directly and negatively regulated by HapR in *V. cholerae* (Gao et al., 2018), thus, we speculated that the transcription of *tcpP*, *toxT*, and *tcpA* might be regulated under the collective effects of HapR and Fur in a subtle manner in *V. cholerae*.

### Transcription of *hapR*, *fur*, *tcpP*, *toxT*, and *tcpA* Were Cell Density-Dependent in the TCP-Induced AKI Culture Condition

**FIGURE 2:**
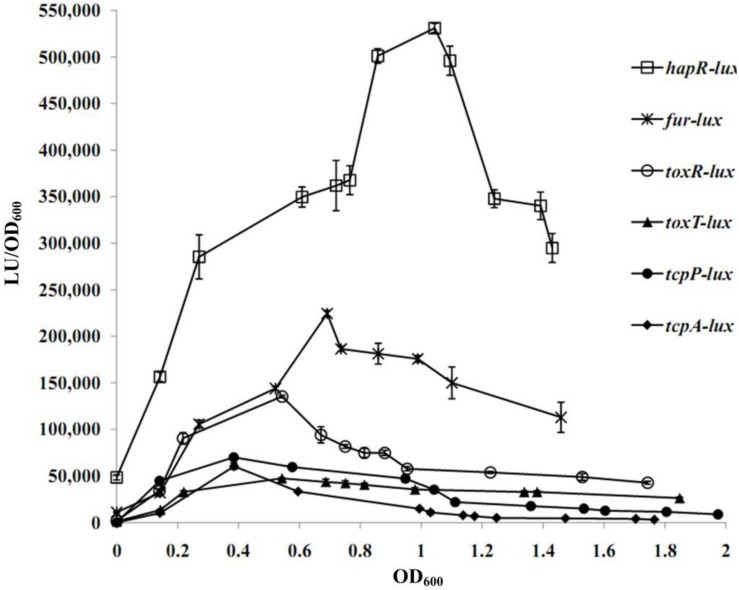
Cell density–dependent expression of target genes. The WT strain was transformed with a recombinant pBBRlux vector that contains a promoter DNA region of the target gene. The bacteria were cultivated in the TCP-induced AKI conditions to determine the luminescence activity under various OD_600_ values.

A transcriptional luminescence reporter assay was applied to detect the transcriptional changes of *hapR*, *fur*, *toxR*, *tcpP*, *toxT*, and *tcpA* during the growth periods of *V. cholerae* in the TCP-induced AKI culture condition. As shown in Figure 2, the transcriptional patterns of all of the genes tested were manifested in a cell-density dependent manner. The highest transcriptional levels of *hapR* and *fur* occurred at an OD_600_ value of around 1.0 and 0.7, respectively, which were consistent with that described in a previous report (Gao et al., 2018). In addition, the transcriptional levels of *tcpP/tcpA* and *toxR*/*toxT* obviously increased with the increase of cell density from 0 to 0.4 and from 0 to 0.6, respectively, and then declined with the further increase of the cell density. The bacteria cells were harvested at the OD_600_ value of about 1.0 and 0.7 for characterizing HapR- and Fur-mediated gene regulation, respectively.

### HapR Repressed Transcription of *tcpP* and *tcpA* Directly, and Repressed *toxT* Indirectly

**FIGURE 3:**
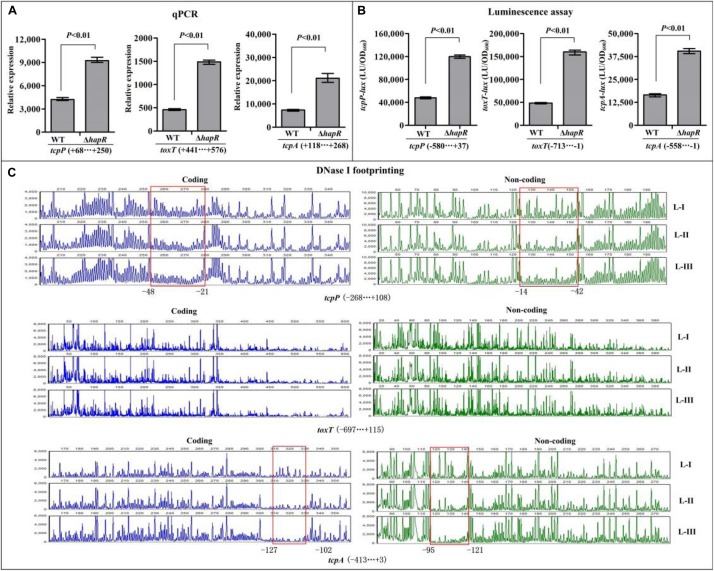
Regulation of *tcpP*, *toxT*, and *tcpA* by HapR. **(A)** qPCR. Relative mRNA levels of the target genes were compared between Δ*hapR* and WT. **(B)** The luminescence assay was done as shown in Figure 2. **(C)** DNase I footprinting. The promoter DNA fragment of each target gene was labeled with FAM and HEX, respectively, and incubated with increasing amounts of purified His-HapR (L-I, -II, and -III contain 0, 2.31, and 6.92 pmol, respectively). After being digested with DNase I, the fragments were analyzed using an ABI 3500XL DNA analyzer. The protected regions are boxed and marked with positions. The negative and positive numbers indicate the nucleotide positions relative to the translation start site (+1) of the corresponding gene.

The qPCR results showed that the mRNA transcription of *tcpP*, *toxT*, and *tcpA* were greatly increased in Δ*hapR* relative to WT (Figure 3A), while that of *toxR* manifested no obvious difference between Δ*hapR* and WT (Supplementary Figure S1A). In addition, the luminescence assays showed that the promoter activities of *tcpP*, *toxT*, and *tcpA* in Δ*hapR* were much higher than that in WT (Figure 3B), whereas that under the control of *toxR* promoter showed a similar magnitude in Δ*hapR* and WT (Supplementary Figure S1B). The DNase I footprinting assay disclosed that His-HapR protected a single DNA region within each of the promoters of *tcpP* and *tcpA*, located from 48 to 14 and 127 to 95 upstream of *tcpP* and *tcpA* against DNase I digestion (Figure 3C), but no HapR binding sites were detected for *toxT* and *toxR* (Figure 3C and Supplementary Figure S1C). Thus, HapR inhibits the transcription of *tcpP* and *tcpA* in a direct manner, but it indirectly represses *toxT* transcription and manifests no regulatory action on *toxR* transcription.

### Fur Directly Activates the Transcriptions of *tcpP*, *toxT*, and *tcpA*

**FIGURE 4:**
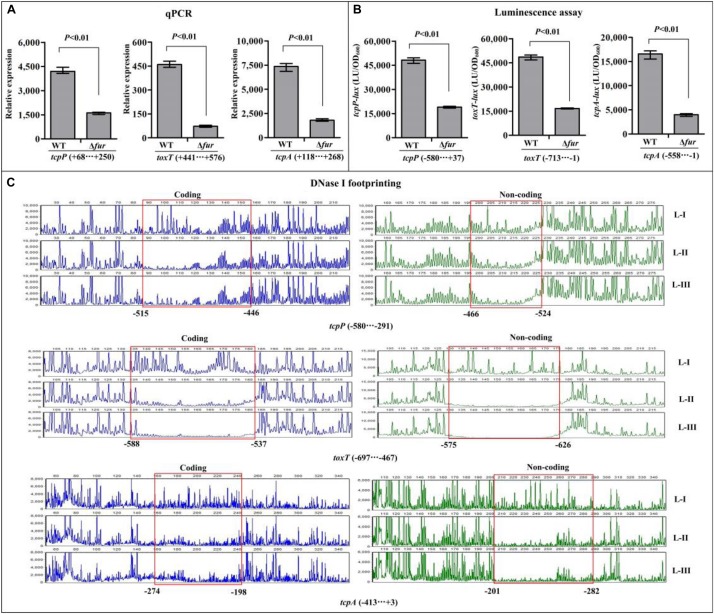
Regulation of *tcpP*, *toxT*, and *tcpA* by Fur. The qPCR **(A)** and DNase I footprinting assays **(C)** were done as Figure 3, while the luminescence assay **(B)** was done as Figure 2. L-I, -II, and -III contain 0, 2.95, and 8.85 pmol of His-Fur, respectively.

The qPCR assay was employed to investigate the regulatory effects of Fur on the transcription of *toxR*, *tcpP*, *toxT*, and *tcpA*, and the results showed that the mRNA levels of *tcpP*, *toxT*, and *tcpA* were obviously decreased in Δ*fur* relative to WT (Figure 4A), while that of *toxR* manifested no obvious difference between Δ*fur* and WT (Supplementary Figure S2A). These results suggested that Fur activates the transcription of *tcpP*, *toxT*, and *tcpA*, but it seems to have no regulatory activity on *toxR* transcription. In addition, the promoter DNA region of *toxR*, *tcpP*, *toxT*, and *tcpA* was each cloned into the pBBRlux plasmid, and then transferred into Δ*fur* and WT, respectively, to test the regulatory actions of Fur on their promoter activities. The results showed that the luminescence under the control of *tcpP*, *toxT*, or *tcpA* promoter in Δ*fur* was much lower than that in WT (Figure 4B), whereas that under the control of *toxR* promoter showed a similar magnitude in Δ*fur* and WT (Supplementary Figure S2B). As further determined by the DNase I footprinting assay, His-Fur protected a single DNA region within each of the promoters of *tcpP*, *toxT*, and *tcpA*, located from 524 to 446, 626 to 537, and 282 to 198 upstream of *tcpP*, *toxT*, and *tcpA* against DNase I digestion in a dose-dependent manner (Figure 4C), but no binding sites were detected for *toxR* (Supplementary Figure S2C). Thus, Fur activates the transcription of *tcpP*, *toxT*, and *tcpA* in a direct manner, but has no regulatory activity on *toxR* transcription.

### Identification of the Transcription Start Site for *tcpA*

The 500 bp upstream DNA regions of *tcpP*, *toxT*, and *tcpA* in El Tor strain C7258 share a high identity (90, 99, and 87%, respectively) in nucleotide sequences with that of in classical biotype strain O395, in which the transcription start sites of these genes have been previously reported (Higgins and DiRita, 1994; Kovacikova and Skorupski, 2001; Goss et al., 2010; Stonehouse et al., 2011) (also seen in Supplementary Figure S3). However, it was notable that the nucleotides in the position of the transcription start site of *tcpA* are different in these two strains (Supplementary Figure S3). Thus, in this study, we mapped the transcription start site of *tcpA* using the primer extension assay. As shown in Supplementary Figure S4, the assay detected only one transcription start site for *tcpA* located at 74 bp upstream of the coding region, the position of which is exactly the same as that of in O395, suggesting that a point mutation in this position has occurred in the two strains. The −10 element is good match with the consensus prokaryotic sequence, but the −35 element is non-conservative, suggesting that *tcpA* possess a relatively weak promoter (Figure 6).

### Fur but Not HapR Plays a Role in Intestinal Colonization of *V. cholerae* in Infant Mice

**FIGURE 5:**
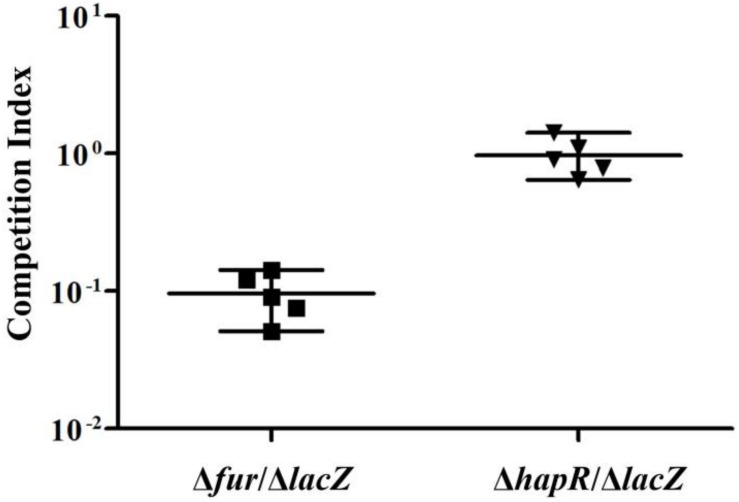
Infant mouse competition assay using Δ*fur*, Δ*hapR*, and Δ*lacZ* cells. The Δ*fur* and Δ*hapR* were competed against Δ*lacZ*. The competitive index is the ratio of mutant to Δ*lacZ* recovered from the intestine calculated by the ratio of input mutant to Δ*lacZ* (inoculated into the mouse). Each data point represents the competitive index from an individual mouse. The line bar represents the geometric mean. The Δ*fur* strain is significantly attenuated (*P* < 0.01) by Student *t*-test.

The *in vivo* competition assay was employed to further investigate the ability of Δ*fur* and Δ*hapR* strains to colonize the small intestine of infant mice in comparison with the Δ*lacZ* strain (Figure 5). The results showed that the colonization ability of Δ*fur* was attenuated approximately 10-fold, while that of Δ*hapR* seemed to have no obvious difference compared to Δ*lacZ* (CI ≈ 1). The same extent colonization capacity of Δ*hapR* as wild-type *V. cholerae* has been previously reported (Zhu et al., 2002; Zhu and Mekalanos, 2003). These results suggested that Fur but not HapR is required for efficient colonization.

## Discussion

*Vibrio cholerae* expresses the virulence determinants to establish colonization in the gut and cause disease diarrhea. Expression of TCP and CT is highly regulated by environmental stimuli and a variety of regulators (Taylor et al., 1987; Miller and Mekalanos, 1988; Nye et al., 2000; Kovacikova and Skorupski, 2001; Zhu et al., 2002; Kovacikova et al., 2004; Mey et al., 2005; Childers and Klose, 2007). The regulatory cascade, which is constituted of ToxR, TcpP, and ToxT referred to as the ToxR virulence regulon, has been shown to be involved in the regulation of TCP and CT, and the regulatory mechanisms also have been well documented (DiRita et al., 1991; Krukonis et al., 2000; Goss et al., 2013). Other regulatory factors modulate TCP and CT production mostly via regulation of the genes within this cascade (Kovacikova and Skorupski, 2001; Kovacikova et al., 2004). HapR also has been shown to be involved in regulating the transcription of *tcpP*, *toxT*, and *tcpA* (Kovacikova and Skorupski, 2002; Zhu et al., 2002), but lacks the direct evidence for its binding to their regulatory regions.

**FIGURE 6:**
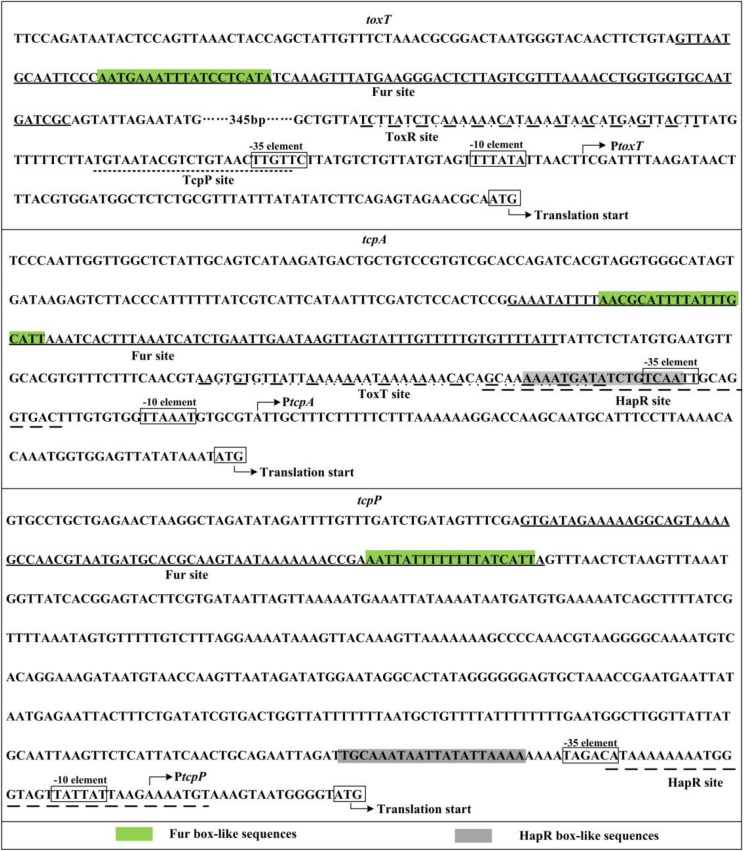
Promoter organization of target genes. The sequences were derived from *Vibrio cholerae* El Tor C7258. The transcription/translation start sites are indicated by bent arrows. The –10 and –35 elements are enclosed in boxes. The binding sites of ToxR and TcpP identified by DNase I digestion were previously reported by Krukonis et al. (2000). The sequence protected by ToxT in the promoter DNA region of *tcpA* was identified by Yu and DiRita (2002) and Withey and DiRita (2006). The Fur sites are underlined with solid lines, whereas the HapR sites are underlined with broken lines.

In the present study, we found a HMRs box-like sequence for each promoter of *tcpP* and *tcpA*, suggesting that the transcription of *tcpP* and *tcpA* would be under the direct control of HapR in *V. cholerae*. We observed that HapR binds to the promoters of *tcpP* and *tcpA* to repress their transcription when the bacterial cells were harvested at an OD_600_ value of about 1.0. The HapR binding site for each *tcpP* and *tcpA* promoter overlaps the core −10 and/or −35 elements, and thus HapR repression of *tcpP* and *tcpA* transcription would be via blocking the entry or elongation of the RNA polymerase. In addition, we noticed that the HapR binding site for *tcpA* promoter partly overlaps with the sequence protected by ToxT (Yu and DiRita, 2002; Withey and DiRita, 2006), which acts as an activator of *tcpA*. Thus, the binding of HapR to the *tcpA* promoter may prevent ToxT from binding to. However, the binding site of HapR for *tcpP* promoter excludes the HMRs box-like sequence, suggesting the results of the informatics analysis are not always reliable. HapR also appears to repress the transcription of *toxT* in an indirect manner, which differs from its interaction with *tcpP* and *tcpA* promoters and could involve additional regulators. A previously study showed that ToxR and TcpP bind adjacently to the promoter DNA region of *toxT* (Figure 6), and the interaction of ToxR and TcpP with the *toxT* promoter enhanced the ability of TcpP to interact with RNA polymerase, leading to transcriptional activation of *toxT* (Krukonis et al., 2000). Thus, HapR repression of *toxT* promoter may be via the directly repression of *tcpP* transcription by HapR.

HapR is a global regulator that controls the expression of hundreds of genes, particularly those responsible for the motility, biofilm formation, metabolism, and virulence (Ball et al., 2017). HapR regulon contains a variety of transcriptional regulator genes, such as *aphA* (Kovacikova and Skorupski, 2002), *vpsT* (Waters et al., 2008), *qstR* (Lo Scrudato and Blokesch, 2013), and *fur* (Gao et al., 2018). The regulatory actions of HapR on other regulatory genes bridge the different regulons in *V. cholerae*, forming complex regulatory networks to tightly control gene expression. For instance, HapR directly regulated the expression of Fur and HlyU, and these regulators coordinately regulated the transcription of *hlyA*, leading to the highest expression level of *hlyA* occurring at the early mid-logarithmic growth phase (Gao et al., 2018). Although the regulatory mechanisms are still obscure, Fur-dependent TCP production has been observed in *V. cholerae* (Mey et al., 2005). Thus, we deduced that expression of TCP is also coordinately regulated by HapR and Fur in *V. cholerae*.

The binding sites of Fur usually contain a 19 bp inverted repeat sequence known as the classic Fur box (Escolar et al., 1999; Mey et al., 2005; Zhou et al., 2006). A 21 bp palindromic sequence was also reported as the enhanced Fur box (Davies et al., 2011), but it shares sequence similarity with the previously predicted (Mey et al., 2005). The Fur box-like sequences were detected for the promoters of *toxT*, *tcpP*, and *tcpA*. Indeed, the transcription of the three above genes was under the direct and positive control of Fur at OD_600_ value of about 0.7. The Fur binding site for each target promoter located far upstream of the core −35 element. Thus, Fur activation of *toxT*, *tcpP*, and *tcpA* transcription may belong to the class I stimulation mechanism (Ishihama, 2000). However, Fur binding sites for *toxT*, *tcpP*, and *tcpA* were not identified by ChIP-seq analysis (Davies et al., 2011). Discrepancies between the present data and the data identified by ChIP-seq could be attributed to the different experimental methods, growth conditions and/or bacterial genetic background, because the ChIP-seq is used to map the binding sites of a regulator *in vivo*. Anyhow, the data presented here showed that Fur activation of TCP production is via at least direct activation of *toxT* and *tcpP* transcription, as well as the TCP coding gene *tcpA*.

Toxin coregulated pilus mostly contributes to the colonization of *V. cholerae* in the host intestine (Kirn et al., 2000). We thus investigated the colonization ability of *fur* and *hapR* deletion mutants in the small intestine of an infant mouse model. The results showed that the colonization ability of Δ*fur* was significantly attenuated relative to that of the Δ*lacZ* strain, which was consistent with previous report (Mey et al., 2005). Fur is an iron-dependent transcriptional regulator that regulates the expression of multitudinous genes involved in iron homeostasis, virulence, biofilm formation, ribosome formation, transporters, porin proteins, and unique sRNAs (Occhino et al., 1998; Davis et al., 2005; Mey et al., 2005; Wyckoff et al., 2007; Craig et al., 2011; Davies et al., 2011; Sun et al., 2012a). Iron is an essential metallic element for life, but there is almost no free iron to use within the intestine of humans at the early infection stage of *V. cholerae* (Ganz, 2018). It was reported that the stools from cholera patients contain a heterogenous mixture of biofilm-like aggregates, and the biofilm formation is initiated almost immediately after adherence of *V. cholerae* to intestinal cells (Faruque et al., 2006; Sengupta et al., 2016). Our unpublished data showed that Δ*fur* produces more biofilms than the WT strain under the iron-starved growth condition. The attenuated colonization ability of Δ*fur* could be attributed to the significantly enhanced biofilm formation and the low production of TCP. The ability of colonization of Δ*hapR* showed no obvious difference compared to that of the Δ*lacZ* strain, the same as previously reported (Zhu et al., 2002). However, the Δ*hapR* strain showed a 10-fold colonization defect relative to the WT strain, when used as the inoculum composed of biofilms to infect infant mice (Zhu and Mekalanos, 2003). The Δ*hapR* strains formed much thicker biofilms, but the detachment of Δ*hapR* from biofilms was defective, which may influence the HapR-dependent colonization in biofilms (Zhu and Mekalanos, 2003). In a word, Fur-dependent but HapR-independent colonization in planktonic cells would be beneficial to the pathogenesis and transmission of *V. cholerae* during the disease progression.

The highest expression levels of *tcpP*, *toxT*, and *tcpA* occurred at an OD_600_ value of around 0.4, but the lower expression levels were observed at both LCD and HCD. AphA is the bottom master regulator of QS that operates at LCD (Lu et al., 2018). However, a previous study showed that AphA binds to the promoter of *tcpPH* to activate its transcription (Kovacikova et al., 2004). TcpP, in turn, activates *toxT* and *tcpA* transcription (DiRita et al., 1991; Krukonis et al., 2000). Thus, we deduced that: at LCD or at the initial stage of infection, AphA activates the expression of TCP and CT via direct activation of *tcpP* expression, which promotes colonization and induces watery diarrhea. However, the intracellular level of AphA protein is rapidly degraded with the increase of cell density (Rutherford et al., 2011). At this point, Fur may be engaged to further activate the expression of TCP through binding and activation of the transcription of *tcpP*, *toxT*, and *tcpA*. While at HCD or at the later stage of infection (high number of *V. cholerae* cells in the intestine), high expressed HapR binds to the promoters of *tcpP* and *tcpA* to repress their transcription, and thus inhibits TCP and CT production, which then enables the pathogen to detach from the epithelium, and exits the host along with the stool. In addition, the transcriptional pattern of *toxR* also manifested a cell-density dependent manner, with a similar results previously observed in *V. cholerae* and the closely related *Vibrio parahaemolyticus* (Xu et al., 2010; Zhang et al., 2017a). However, both HapR and Fur seemed to have no regulatory actions on *toxR* transcription under the current growth conditions. The molecular mechanism of cell-density dependent expression of *toxR*, therefore, needs to be further investigated. Nevertheless, the present work enriched the virulence regulatory networks in *V. cholerae*, and broadens our understanding of the pathogenic mechanisms of the pathogen.

## Data Availability Statement

The raw data supporting the conclusions of this article will be made available by the authors, without undue reservation, to any qualified researcher.

## Ethics Statement

The animal study was reviewed and approved by the Ethics Committee of the National Institute for Communicable Disease Control and Prevention, China CDC. Written informed consent was obtained from the owners for the participation of their animals in this study.

## Author Contributions

HG, YZ, and BK conceived the study and designed experimental procedures. JZ, JLo, JLi, QQ, and QS performed the experiments and carried out data analysis. HG, YZ, and BK wrote the manuscript.

## Conflict of Interest

The authors declare that the research was conducted in the absence of any commercial or financial relationships that could be construed as a potential conflict of interest.

## Footnotes

1 http://embnet.ccg.unam.mx/rsat/

## Supplementary Material

FIGURE S1 Regulation of *toxR* by HapR. The qPCR **(A)** and DNase I footprinting assays were done as in Figure 3, while the luminescence assay **(B)** was done as in Figure 2. L-I, -II, and -III contain 0, 2.31, and 6.92 pmol of His-HapR, respectively.

FIGURE S2 Regulation of *toxR* by Fur. The qPCR **(A)** and DNase I footprinting assays were done as Figure 3, while the luminescence assay **(B)** was done as in Figure 2. L-I, -II, and -III contain 0, 2.95, and 8.85 pmol of His-Fur, respectively.

FIGURE S3 The nucleotide sequences alignment of the promoter DNA regions of *tcpP* and *toxT*. The promoter DNA sequences of *tcpP*, *toxT* and *tcpA* were derived from *V. cholerae* El Tor biotype strain C7258 and classical biotype strain O395. The different bases were labeledred, while the identical bases were marked with asterisk (*). Shown also were the transcription start sites, -10 and -35 boxes.

FIGURE S4 Transcription start site of *tcpA* in *V. cholerae* El Tor biotype strain C7258. A 5′-HEX-labeled reverse primer was designed to be complementary to the RNA transcript of *tcpA*. The primer extension products were analyzed with an ABI 3500XL DNA Genetic analyzer. The transcription start site was marked with asterisks andpositions.

## References

[B1] Abuaita B. H. Withey J. H. (2011). Termination of *Vibrio cholerae* virulence gene expression is mediated by proteolysis of the major virulence activator, ToxT. *Mol. Microbiol.* 81 1640–1653. 10.1111/j.1365-2958.2011.07798.x 21883522

[B2] Ball A. S. Chaparian R. R. van Kessel J. C. (2017). Quorum sensing gene regulation by LuxR/HapR master regulators in *Vibrios*. *J. Bacteriol.* 199 e105–e117. 10.1128/JB.00105-17 28484045 PMC5585708

[B3] Childers B. M. Klose K. E. (2007). Regulation of virulence in *Vibrio cholerae*: the ToxR regulon. *Future Microbiol.* 2 335–344. 10.2217/17460913.2.3.335 17661707

[B4] Clemens J. D. Nair G. B. Ahmed T. Qadri F. Holmgren J. (2017). Cholera. *Lancet* 390 1539–1549. 10.1016/S0140-6736(17)30559-7 28302312

[B5] Craig S. A. Carpenter C. D. Mey A. R. Wyckoff E. E. Payne S. M. (2011). Positive regulation of the *Vibrio cholerae* porin OmpT by iron and fur. *J. Bacteriol.* 193 6505–6511. 10.1128/JB.05681-11 21965571 PMC3232907

[B6] Davies B. W. Bogard R. W. Mekalanos J. J. (2011). Mapping the regulon of *Vibrio cholerae* ferric uptake regulator expands its known network of gene regulation. *Proc. Natl. Acad. Sci. U.S.A.* 108 12467–12472. 10.1073/pnas.1107894108 21750152 PMC3145737

[B7] Davis B. M. Quinones M. Pratt J. Ding Y. Waldor M. K. (2005). Characterization of the small untranslated RNA RyhB and its regulon in *Vibrio cholerae*. *J. Bacteriol.* 187 4005–4014. 10.1128/JB.187.12.4005-4014.2005 15937163 PMC1151736

[B8] DiRita V. J. Parsot C. Jander G. Mekalanos J. J. (1991). Regulatory cascade controls virulence in *Vibrio cholerae*. *Proc. Natl. Acad. Sci. U.S.A.* 88 5403–5407. 10.1073/pnas.88.12.5403 2052618 PMC51881

[B9] Escolar L. Perez-Martin J. de Lorenzo V. (1999). Opening the iron box: transcriptional metalloregulation by the Fur protein. *J. Bacteriol.* 181 6223–6229. 10.1128/jb.181.20.6223-6229.1999 10515908 PMC103753

[B10] Faruque S. M. Biswas K. Udden S. M. Ahmad Q. S. Sack D. A. Nair G. B. (2006). Transmissibility of cholera: *in vivo*-formed biofilms and their relationship to infectivity and persistence in the environment. *Proc. Natl. Acad. Sci. U.S.A.* 103 6350–6355. 10.1073/pnas.0601277103 16601099 PMC1458881

[B11] Ganz T. (2018). Iron and infection. *Int. J. Hematol.* 107 7–15. 10.1007/s12185-017-2366-2 29147843

[B12] Gao H. Xu J. Lu X. Li J. Lou J. Zhao H. (2018). Expression of hemolysin is regulated under the collective actions of HapR, Fur, and HlyU in *Vibrio cholerae* El Tor serogroup O1. *Front. Microbiol.* 9:1310. 10.3389/fmicb.2018.01310 29971055 PMC6018088

[B13] Gao H. Zhang L. Osei-Adjei G. Yang W. Zhou D. Huang X. (2017). Transcriptional regulation of Q-mfpABC and mfpABC by CalR in *Vibrio parahaemolyticus*. *Microbiologyopen* 6:e00470. 10.1002/mbo3.470 28318117 PMC5552913

[B14] Gao H. Zhang Y. Yang L. Liu X. Guo Z. Tan Y. (2011). Regulatory effects of cAMP receptor protein (CRP) on porin genes and its own gene in *Yersinia pestis*. *BMC Microbiol.* 11:40. 10.1186/1471-2180-11-40 21345179 PMC3050693

[B15] Gao H. Zhou D. Li Y. Guo Z. Han Y. Song Y. (2008). The iron-responsive Fur regulon in *Yersinia pestis*. *J. Bacteriol.* 190 3063–3075. 10.1128/JB.01910-07 18281395 PMC2293247

[B16] Goss T. J. Morgan S. J. French E. L. Krukonis E. S. (2013). ToxR recognizes a direct repeat element in the toxT, ompU, ompT, and ctxA promoters of *Vibrio cholerae* to regulate transcription. *Infect. Immun.* 81 884–895. 10.1128/IAI.00889-12 23297386 PMC3584884

[B17] Goss T. J. Seaborn C. P. Gray M. D. Krukonis E. S. (2010). Identification of the TcpP-binding site in the toxT promoter of *Vibrio cholerae* and the role of ToxR in TcpP-mediated activation. *Infect. Immun.* 78 4122–4133. 10.1128/IAI.00566-10 20679441 PMC2950353

[B18] Henke J. M. Bassler B. L. (2004). Three parallel quorum-sensing systems regulate gene expression in *Vibrio harveyi*. *J. Bacteriol.* 186 6902–6914. 10.1128/JB.186.20.6902-6914.2004 15466044 PMC522208

[B19] Higgins D. E. DiRita V. J. (1994). Transcriptional control of toxT, a regulatory gene in the ToxR regulon of *Vibrio cholerae*. *Mol. Microbiol.* 14 17–29. 10.1111/j.1365-2958.1994.tb01263.x 7830555

[B20] Hung D. T. Mekalanos J. J. (2005). Bile acids induce cholera toxin expression in *Vibrio cholerae* in a ToxT-independent manner. *Proc. Natl. Acad. Sci. U.S.A.* 102 3028–3033. 10.1073/pnas.0409559102 15699331 PMC549475

[B21] Ishihama A. (2000). Functional modulation of *Escherichia coli* RNA polymerase. *Annu. Rev. Microbiol.* 54 499–518. 10.1146/annurev.micro.54.1.499 11018136

[B22] Iwanaga M. Yamamoto K. Higa N. Ichinose Y. Nakasone N. Tanabe M. (1986). Culture conditions for stimulating cholera toxin production by *Vibrio cholerae* O1 El Tor. *Microbiol. Immunol.* 30 1075–1083. 10.1111/j.1348-0421.1986.tb03037.x 3543624

[B23] Kirn T. J. Lafferty M. J. Sandoe C. M. Taylor R. K. (2000). Delineation of pilin domains required for bacterial association into microcolonies and intestinal colonization by *Vibrio cholerae*. *Mol. Microbiol.* 35 896–910. 10.1046/j.1365-2958.2000.01764.x 10692166

[B24] Kleber-Janke T. Becker W. M. (2000). Use of modified BL21(DE3) *Escherichia coli* cells for high-level expression of recombinant peanut allergens affected by poor codon usage. *Protein Express. Purif.* 19 419–424. 10.1006/prep.2000.1265 10910733

[B25] Kovacikova G. Lin W. Skorupski K. (2004). *Vibrio cholerae* AphA uses a novel mechanism for virulence gene activation that involves interaction with the LysR-type regulator AphB at the tcpPH promoter. *Mol. Microbiol.* 53 129–142. 10.1111/j.1365-2958.2004.04121.x 15225309

[B26] Kovacikova G. Skorupski K. (2001). Overlapping binding sites for the virulence gene regulators AphA, AphB and cAMP-CRP at the *Vibrio cholerae* tcpPH promoter. *Mol. Microbiol.* 41 393–407. 10.1046/j.1365-2958.2001.02518.x 11489126

[B27] Kovacikova G. Skorupski K. (2002). Regulation of virulence gene expression in *Vibrio cholerae* by quorum sensing: HapR functions at the aphA promoter. *Mol. Microbiol.* 46 1135–1147. 10.1046/j.1365-2958.2002.03229.x 12421317

[B28] Krukonis E. S. Yu R. R. Dirita V. J. (2000). The *Vibrio cholerae* ToxR/TcpP/ToxT virulence cascade: distinct roles for two membrane-localized transcriptional activators on a single promoter. *Mol. Microbiol.* 38 67–84. 10.1046/j.1365-2958.2000.02111.x 11029691

[B29] Lee H. J. Bang S. H. Lee K. H. Park S. J. (2007). Positive regulation of fur gene expression via direct interaction of fur in a pathogenic bacterium, *Vibrio vulnificus*. *J. Bacteriol.* 189 2629–2636. 10.1128/JB.01791-06 17237166 PMC1855807

[B30] Lo Scrudato M. Blokesch M. (2013). A transcriptional regulator linking quorum sensing and chitin induction to render *Vibrio cholerae* naturally transformable. *Nucleic Acids Res.* 41 3644–3658. 10.1093/nar/gkt041 23382174 PMC3616704

[B31] Lu R. Osei-Adjei G. Huang X. Zhang Y. (2018). Role and regulation of the orphan AphA protein of quorum sensing in pathogenic *Vibrios*. *Future Microbiol.* 13 383–391. 10.2217/fmb-2017-0165 29441822

[B32] Matson J. S. DiRita V. J. (2005). Degradation of the membrane-localized virulence activator TcpP by the YaeL protease in *Vibrio cholerae*. *Proc. Natl. Acad. Sci. U.S.A.* 102 16403–16408. 10.1073/pnas.0505818102 16254052 PMC1283431

[B33] Merritt E. A. Hol W. G. (1995). AB5 toxins. *Curr. Opin. Struct Biolo.* 5 165–171. 10.1016/0959-440x(95)80071-9 7648317

[B34] Mey A. R. Wyckoff E. E. Kanukurthy V. Fisher C. R. Payne S. M. (2005). Iron and fur regulation in *Vibrio cholerae* and the role of fur in virulence. *Infect. Immun.* 73 8167–8178. 10.1128/IAI.73.12.8167-8178.2005 16299312 PMC1307094

[B35] Miller M. B. Skorupski K. Lenz D. H. Taylor R. K. Bassler B. L. (2002). Parallel quorum sensing systems converge to regulate virulence in *Vibrio cholerae*. *Cell* 110 303–314. 10.1016/s0092-8674(02)00829-2 12176318

[B36] Miller V. L. Mekalanos J. J. (1988). A novel suicide vector and its use in construction of insertion mutations: osmoregulation of outer membrane proteins and virulence determinants in *Vibrio cholerae*. *J. Bacteriol.* 170 2575–2583. 10.1128/jb.170.6.2575-2583.1988 2836362 PMC211174

[B37] Nye M. B. Pfau J. D. Skorupski K. Taylor R. K. (2000). *Vibrio cholerae* H-NS silences virulence gene expression at multiple steps in the ToxR regulatory cascade. *J. Bacteriol.* 182 4295–4303. 10.1128/jb.182.15.4295-4303.2000 10894740 PMC101945

[B38] Occhino D. A. Wyckoff E. E. Henderson D. P. Wrona T. J. Payne S. M. (1998). *Vibrio cholerae* iron transport: haem transport genes are linked to one of two sets of tonB, exbB, exbD genes. *Mol. Microbiol.* 29 1493–1507. 10.1046/j.1365-2958.1998.01034.x 9781885

[B39] Rutherford S. T. van Kessel J. C. Shao Y. Bassler B. L. (2011). AphA and LuxR/HapR reciprocally control quorum sensing in *Vibrios*. *Genes Dev.* 25 397–408. 10.1101/gad.2015011 21325136 PMC3042162

[B40] Sengupta C. Mukherjee O. Chowdhury R. (2016). Adherence to Intestinal Cells Promotes Biofilm Formation in *Vibrio cholerae*. *J. Infect. Dis.* 214 1571–1578. 10.1093/infdis/jiw435 27638940

[B41] Stonehouse E. A. Hulbert R. R. Nye M. B. Skorupski K. Taylor R. K. (2011). H-NS binding and repression of the ctx promoter in *Vibrio cholerae*. *J. Bacteriol.* 193 979–988. 10.1128/JB.01343-09 21169492 PMC3028689

[B42] Sun F. Gao H. Zhang Y. Wang L. Fang N. Tan Y. (2012a). Fur is a repressor of biofilm formation in *Yersinia pestis*. *PLoS One* 7:e52392. 10.1371/journal.pone.0052392 23285021 PMC3528687

[B43] Sun F. Zhang Y. Wang L. Yan X. Tan Y. Guo Z. (2012b). Molecular characterization of direct target genes and cis-acting consensus recognized by quorum-sensing regulator AphA in *Vibrio parahaemolyticus*. *PLoS One* 7:e44210. 10.1371/journal.pone.0044210 22984476 PMC3440409

[B44] Tamayo R. Patimalla B. Camilli A. (2010). Growth in a biofilm induces a hyperinfectious phenotype in *Vibrio cholerae*. *Infect. Immun.* 78 3560–3569. 10.1128/IAI.00048-10 20515927 PMC2916270

[B45] Taylor R. K. Miller V. L. Furlong D. B. Mekalanos J. J. (1987). Use of phoA gene fusions to identify a pilus colonization factor coordinately regulated with cholera toxin. *Proc. Natl. Acad. Sci. U.S.A.* 84 2833–2837. 10.1073/pnas.84.9.2833 2883655 PMC304754

[B46] Tu K. C. Long T. Svenningsen S. L. Wingreen N. S. Bassler B. L. (2010). Negative feedback loops involving small regulatory RNAs precisely control the *Vibrio harveyi* quorum-sensing response. *Mol. Cell* 37 567–579. 10.1016/j.molcel.2010.01.022 20188674 PMC2844700

[B47] Waldor M. K. Mekalanos J. J. (1996). Lysogenic conversion by a filamentous phage encoding cholera toxin. *Science* 272 1910–1914. 10.1126/science.272.5270.1910 8658163

[B48] Waters C. M. Lu W. Rabinowitz J. D. Bassler B. L. (2008). Quorum sensing controls biofilm formation in *Vibrio cholerae* through modulation of cyclic di-GMP levels and repression of vpsT. *J. Bacteriol.* 190 2527–2536. 10.1128/JB.01756-07 18223081 PMC2293178

[B49] Withey J. H. DiRita V. J. (2006). The toxbox: specific DNA sequence requirements for activation of *Vibrio cholerae* virulence genes by ToxT. *Mol. Microbiol.* 59 1779–1789. 10.1111/j.1365-2958.2006.05053.x 16553883

[B50] Wu R. Zhao M. Li J. Gao H. Kan B. Liang W. (2015). Direct regulation of the natural competence regulator gene tfoX by cyclic AMP (cAMP) and cAMP receptor protein (CRP) in *Vibrios*. *Sci. Rep.* 5:14921. 10.1038/srep14921 26442598 PMC4595672

[B51] Wyckoff E. E. Mey A. R. Payne S. M. (2007). Iron acquisition in *Vibrio cholerae*. *Biometals* 20 405–416. 10.1007/s10534-006-9073-4 17216354

[B52] Xu X. Stern A. M. Liu Z. Kan B. Zhu J. (2010). Virulence regulator AphB enhances toxR transcription in *Vibrio cholerae*. *BMC Microbiol.* 10:3. 10.1186/1471-2180-10-3 20053280 PMC2806343

[B53] Yu R. R. DiRita V. J. (2002). Regulation of gene expression in *Vibrio cholerae* by ToxT involves both antirepression and RNA polymerase stimulation. *Mol. Microbiol.* 43 119–134. 10.1046/j.1365-2958.2002.02721.x 11849541

[B54] Zhang Y. Gao H. Osei-Adjei G. Zhang Y. Yang W. Yang H. (2017a). Transcriptional regulation of the type VI secretion system 1 genes by quorum sensing and ToxR in *Vibrio parahaemolyticus*. *Front. Microbiol.* 8:2005 10.3389/fmicb.2017.02005 PMC5650642 29085350

[B55] Zhang Y. Zhang Y. Gao H. Zhang L. Yin Z. Huang X. (2017b). *Vibrio parahaemolyticus* CalR down regulates the thermostable direct hemolysin (TDH) gene transcription and thereby inhibits hemolytic activity. *Gene* 613 39–44. 10.1016/j.gene.2017.03.001 28268179

[B56] Zhang Y. Qiu Y. Tan Y. Guo Z. Yang R. Zhou D. (2012). Transcriptional regulation of opaR, qrr2-4 and aphA by the master quorum-sensing regulator OpaR in *Vibrio parahaemolyticus*. *PLoS One* 7:e34622. 10.1371/journal.pone.0034622 22506036 PMC3323551

[B57] Zhou D. Qin L. Han Y. Qiu J. Chen Z. Li B. (2006). Global analysis of iron assimilation and fur regulation in *Yersinia pestis*. *FEMS Microbiol. Lett.* 258 9–17. 10.1111/j.1574-6968.2006.00208.x 16630248

[B58] Zhu J. Mekalanos J. J. (2003). Quorum sensing-dependent biofilms enhance colonization in *Vibrio cholerae*. *Dev. Cell* 5 647–656. 10.1016/s1534-5807(03)00295-8 14536065

[B59] Zhu J. Miller M. B. Vance R. E. Dziejman M. Bassler B. L. Mekalanos J. J. (2002). Quorum-sensing regulators control virulence gene expression in *Vibrio cholerae*. *Proc. Natl. Acad. Sci. U.S.A.* 99 3129–3134. 10.1073/pnas.052694299 11854465 PMC122484

