# Supplementary figures and images for: Direct Binding and Regulation by Fur and HapR of the Intermediate Regulator and Virulence Factor Genes Within the ToxR Virulence Regulon in Vibrio cholerae
Source: Front Microbiol. 2020 Apr 17;11:709. doi: 10.3389/fmicb.2020.00709 (PMC7181404; doi:10.3389/fmicb.2020.00709)

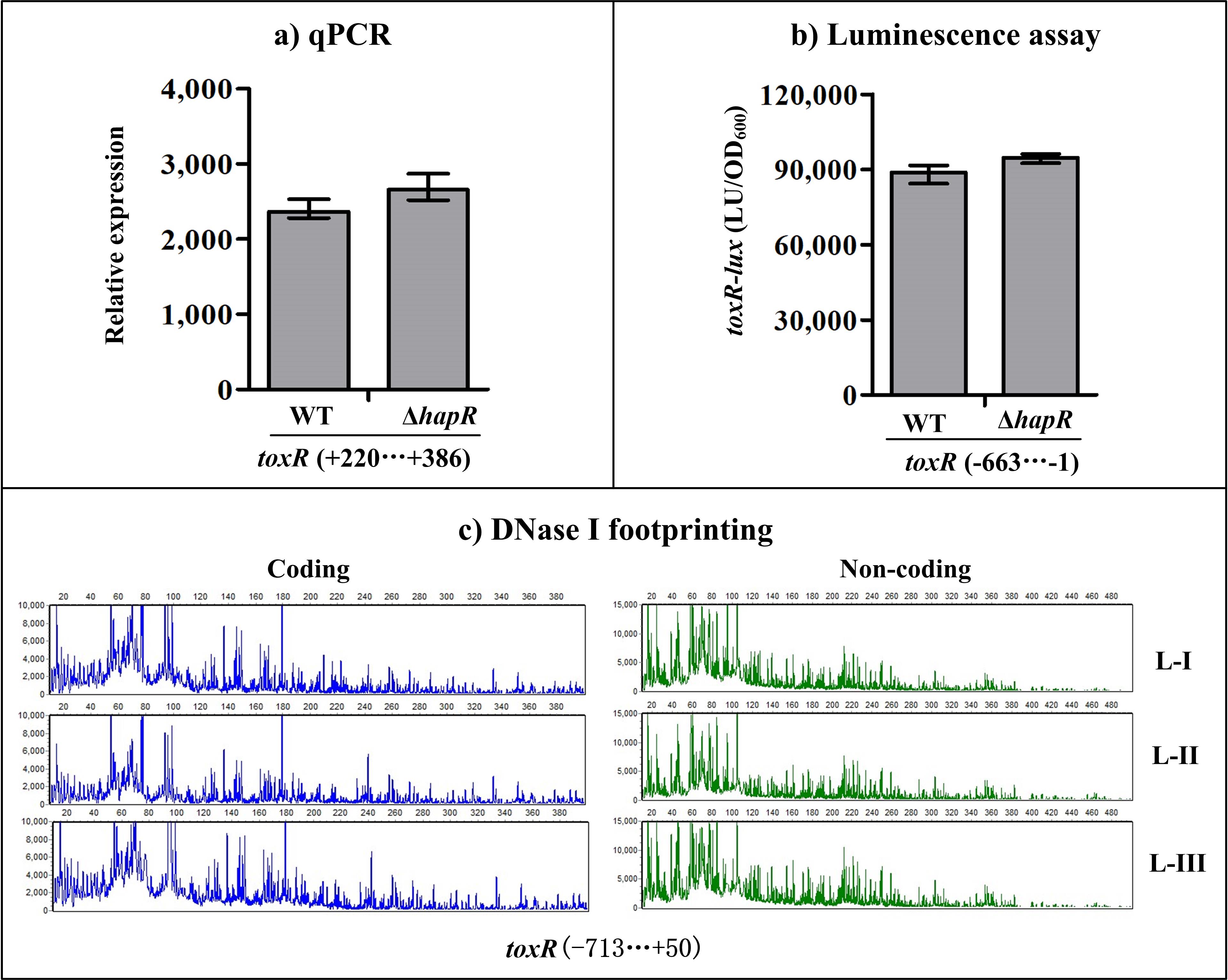

Supplement: FIGURE S1 — Regulation of toxR by HapR. The qPCR (A) and DNase I footprinting assays were done as in Figure 3, while the luminescence assay (B) was done as in Figure 2. L-I, -II, and -III contain 0, 2.31, and 6.92 pmol of His-HapR, respectively. [file Image_1.jpg]

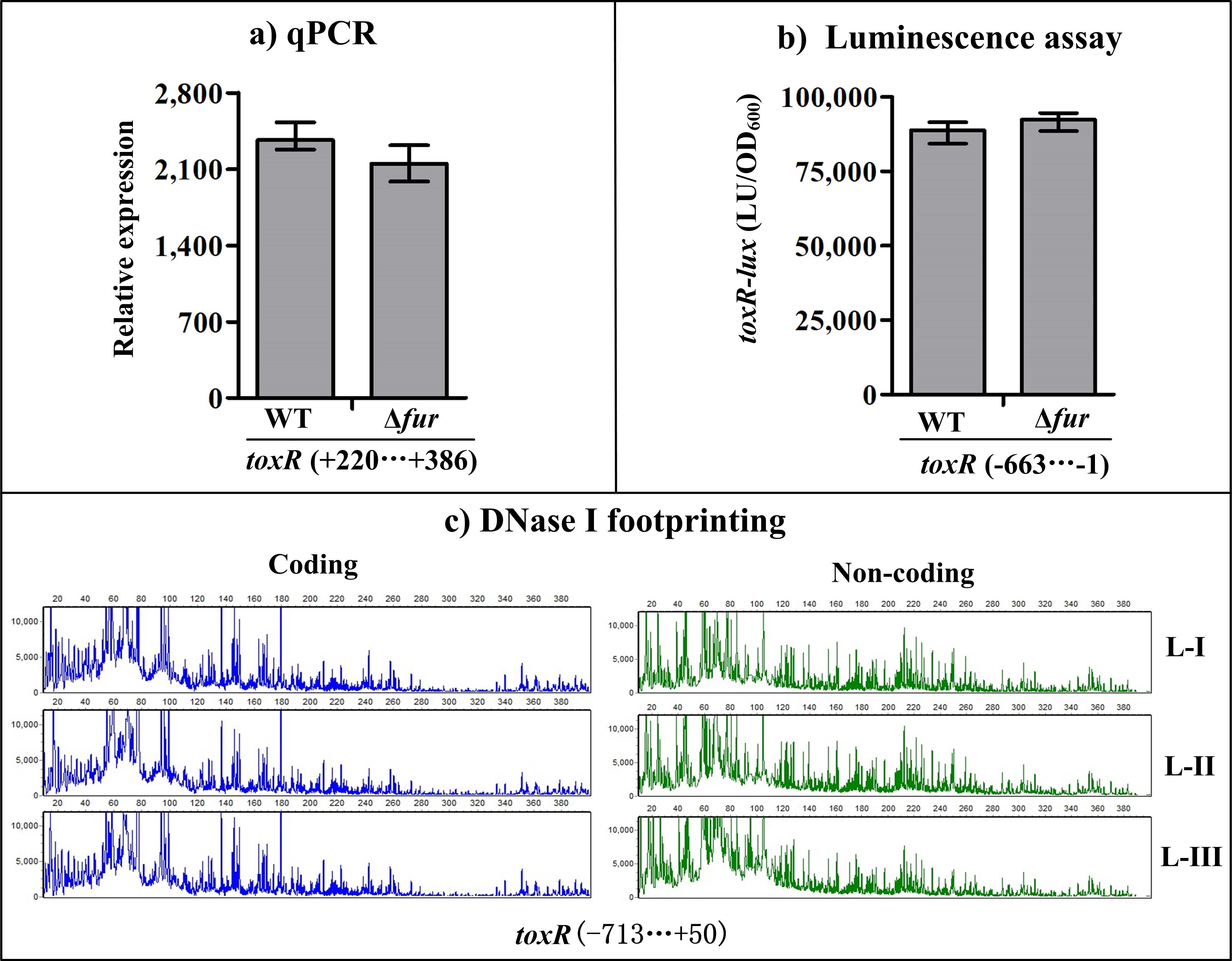

Supplement: FIGURE S2 — Regulation of toxR by Fur. The qPCR (A) and DNase I footprinting assays were done as Figure 3, while the luminescence assay (B) was done as in Figure 2. L-I, -II, and -III contain 0, 2.95, and 8.85 pmol of His-Fur, respectively. [file Image_2.jpg]

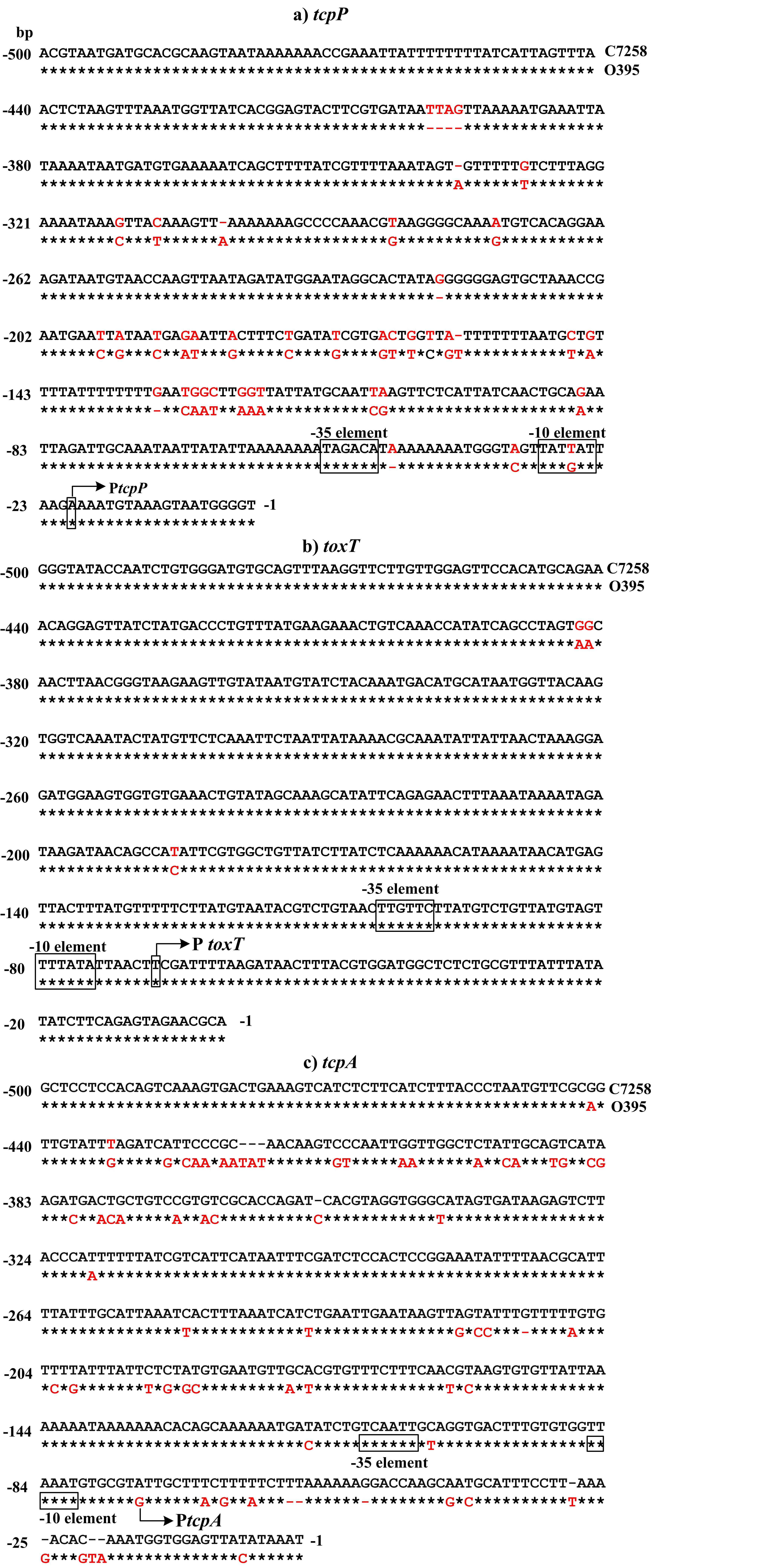

Supplement: FIGURE S3 — The nucleotide sequences alignment of the promoter DNA regions of tcpP and toxT. The promoter DNA sequences of tcpP, toxT and tcpA were derived from V. cholerae El Tor biotype strain C7258 and classical biotype strain O395. The different bases were labeledred, while the identical bases were marked with asterisk (*). Shown also were the transcription start sites, -10 and -35 boxes. [file Image_3.jpg]

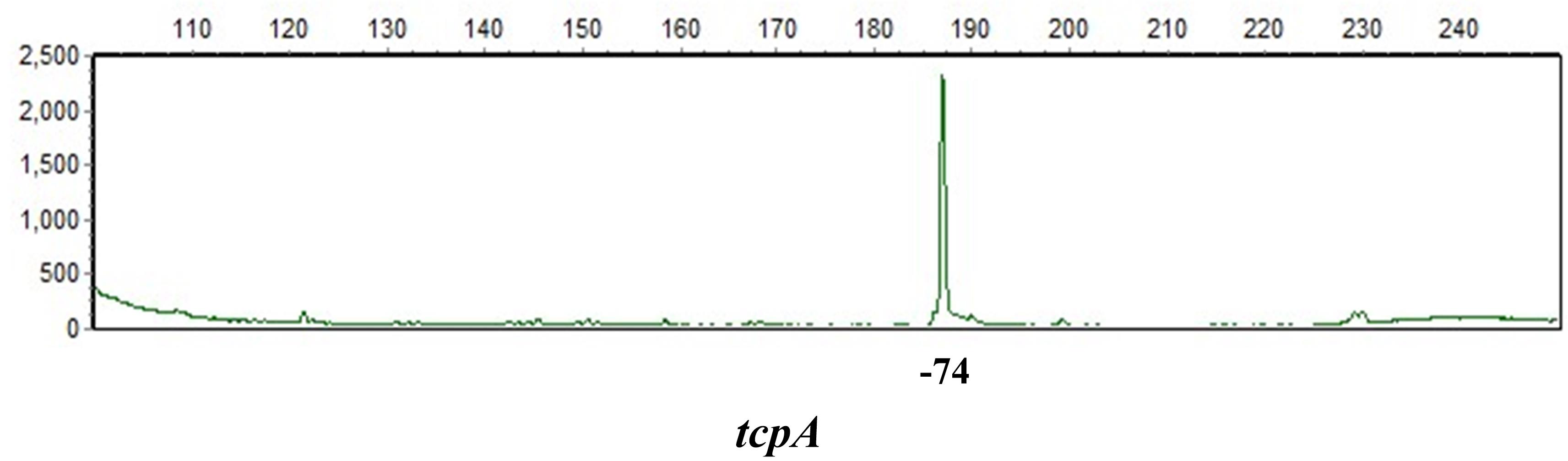

Supplement: FIGURE S4 — Transcription start site of tcpA in V. cholerae El Tor biotype strain C7258. A 5′-HEX-labeled reverse primer was designed to be complementary to the RNA transcript of tcpA. The primer extension products were analyzed with an ABI 3500XL DNA Genetic analyzer. The transcription start site was marked with asterisks andpositions. [file Image_4.jpg]
